# “Back to a false normality”: new intriguing mechanisms of resistance to PARP inhibitors

**DOI:** 10.18632/oncotarget.14409

**Published:** 2016-12-31

**Authors:** Lorena Incorvaia, Francesc Passiglia, Sergio Rizzo, Antonio Galvano, Angela Listȶ, Nadia Barraco, Rossella Maragliano, Valentina Calò, Clara Natoli, Marcello Ciaccio, Viviana Bazan, Antonio Russo

**Affiliations:** ^1^ Department of Surgical, Oncological and Oral Sciences, University of Palermo, Palermo, Italy; ^2^ Department of Medical, Oral and Biotechnological Sciences, Centre of Ageing Sciences and Translational Medicine - CESI-MeT University “G. D'Annunzio”, Chieti, Italy; ^3^ Section of Clinical Biochemistry and Clinical Molecular Medicine, Department of Biopathology and Medical Biotechnology, University of Palermo - U.O.C. Laboratory Medicine - CoreLab, Policlinico University Hospital, Palermo, Italy

**Keywords:** PARP inhibitors, BRCA1-2, resistance

## Abstract

Several evidences have shown that BRCA mutations increased tumor-cells sensitivity to PARP inhibitors by synthetic lethality leading to an accelerated development of several compounds targeting the PARP enzymes system as anticancer agents for clinical setting. Most of such compounds have been investigated in ovarian and breast cancer, showing promising efficacy in BRCA-mutated patients. Recently clinical studies of PARP-inhibitors have been extended across different tumor types harboring BRCA-mutations, including also “BRCA-like” sporadic tumors with homologous recombination deficiency (HRD). This review summarizes the biological background underlying PARP-inhibition, reporting the results of the most relevant clinical trials carried out in patients treated with PARP inhibitors alone or in combination with chemotherapy. Molecular mechanisms responsible for the occurrence of both primary and acquired resistance have been elucidated, in order to support the development of new treatment strategies.

## INTRODUCTION

The personalized therapeutic strategy targeting specific molecular alterations involved into tumorigenesis is the new frontier of cancer treatment. Dysfunction of DNA damage repair from cancer, impairing the integrity of the genome, could be a valuable target for anticancer therapy.

BRCA germline mutations represent the molecular basis for the majority of hereditary breast and ovarian cancer (HBOC) syndrome. BRCA1 and BRCA2 are tumor suppressor genes encoding proteins essential for DNA repair, involved in key processes for the maintenance of genomic stability [1].

Specifically BRCA1/2 are essential for the accurate repair of DNA double-strand breaks (DSBs) through homologous recombination (HR) [2]. This mechanism of DNA repair is conservative and it allows to restore the original sequence of the DNA [3].

Mutations in BRCA1 or BRCA2 result in protein isoforms unable to repair DSBs by HR. The HR-deficient cells use alternative DNA-damage response (DDR) pathway. This compensatory repair mechanism is non-conservative, causes genomic instability and make the tumor cells subordinated to their proper functioning to survival [2, 3].

Germline mutations in tumor suppressors BRCA1/2 result in an increased risk of developing breast, ovarian and others, such as pancreas, stomach and prostate cancers [4, 5]: the neoplastic process will rise if the wild-type gene is lost or inactivated.

Other genes and low penetrance alleles can be involved in HBOC susceptibility including some genes encoding for proteins involved in HR DNA repair pathway, such as RAD50, RAD51C, RAD51D, PALB2, CHEK2, MRE11A, BARD1, BRIP 1, NBS1 and ATM [3]. Furthermore alterations in such genes have been identified also in sporadic breast tumors with early age of onset, hormone receptors (ER/PgR) negativity and a high tumor grade, but in absence of germline BRCA1/2 mutations, which define a BRCA-like phenotype defined as “BRCAness” [5].

All these findings suggest that DNA repair process and specially the HR are associated with the onset of many tumors and have today important therapeutic implications for patients: the loss of a functional copy of BRCA1/2 is a workable target for using a synthetic lethal approach to treat BRCA-deficient cancers [6].

The concept of synthetic lethality between two genes imply that the concomitant damage of both produces the cell death. Conversely, the loss of either gene allows cell survival. BRCA1/2 and Poly(ADPribose) polymerase 1 (PARP1) genes are a model of synthetic lethality [7]. PARP1 encodes an enzyme with an important role in DNA single-strand break repair (SSBs) [8].

PARP inhibitors (PARPi), an emerging class of drugs, are synthetically lethal against BRCA1/2-deficient cancer cells. In these cells the PARP inhibition produces accumulation of genetic aberrations that cannot be restored through BRCA1/2-homologous recombination [9]. The PARPi target is, therefore, the dysregulation of DNA repair pathway and the subsequent addiction of the BRCA-mutated tumors by the function of compensatory repair mechanism.

PARPi have been developed and tested in clinical trials to treat patients carrying BRCA1/2 mutations. These genetic alterations have been detected in about 17% of patients with ovarian cancer, mainly high grade serous adenocarcinoma. These findings prompted the approval of the first PARPi olaparib in Dicember 2014 as monotherapy for the treatment of patients with advanced ovarian cancer and BRCA1/2 germline mutations who have received three or more prior lines of chemotherapy [10–12].

Currently, several clinical trials are investigating the effectiveness of new PARPi, alone or in combination with chemotherapy or targeted therapy [13]. Moreover, the discovery of somatic BRCA mutations also in other cancer provides new and promising applications for PARPi.

Most data from clinical trials suggests that some patients initially treated with PARPi eventually develop resistance [14]. The mechanisms of resistance are multiple and they typically restore the function of DDR pathway [15, 16].

## BIOLOGY OF PARP INHIBITION

Several genotoxic agents, including reactive oxygen species, ultraviolet light, ionizing radiation, and endogenous and synthetic compounds may lead to single strand break (SSB) or double strands breaks (DSB) at the DNA helix, which are physiologically repaired by different systems that provide the restoration and maintenance of DNA integrity [17].

During replication (cell cycle S phase) polymerases usually replace the incorrect single nucleotides insertions by proofreading and exonuclease activity, whereas if the new synthesized strand presents deletions, insertions or wrong bases incorporations, mismatch repair mechanisms (MMR) will be activated.

Both polymerases and MMR are able to repair only “simple” replication errors consisting of single nucleotides alterations. If more “complex alterations” occur, such as chromosomal rearrangements and transposons, the final effect will be the DNA damage.

In this case the cells put in place two main strategies aiming to correct these negative events: first stopping the cell cycle, and subsequently activating the repair cellular pathways. If the damage cannot be corrected cells will die by apoptosis. After the detection of DNA lesions several check-point proteins are recruited, including DNA-dependent protein kinase (DNA-PK), ataxia-telangiectasia mutated (ATM) and and Rad3-related kinase (ATR). All these are serine threonine-kinase enzymes responsible for the activation of specific damage-related repair systems during the check-point response. The activation of repair mechanisms is correlated to the specific DNA damage causes and extent.

The two main cellular systems responsible for the correction of the SSB are called: Base Excision Repair (BER) and Nucleotide Excision Repair (NER).

BER occurs after X rays, oxygen radicals, alkylating agents or spontaneous reactions which alter the nucleotide bases during S and G2 phases; while NER complex is activated by UV light, which arises pyridine dimers or large DNA adducts, and has an important role during G1 phase [18, 19].

BER system activity is initiated by a DNA glycosylase which recognizes the damaged site and removes the nucleotide base; after that, X-ray repair cross-complementing gene 1 (XRCC1) protein, DNA polymerase B (polβ), exo and endo-nucleases enzymes are recruited and provide to the repair, synthesis and ligation of DNA [20, 21]. Another enzyme involved in the BER pathway, is the poly ADP-ribose polymerases (PARP). PARP is a post-translation protein that synthesizes ADP-ribose polymers (PAR) on target proteins by the nicotinamide adenine dinucleotide NAD+ substrate, facilitating the annealing of system repairs. These enzymes have a pleiotropic function in response to genotoxic stressors aiming to maintain the genomic stability. They take part to different processes: cellular metabolism and death, chromatin modification, insulator function, mitotic apparatus response, and transcriptional regulation. PARP1 was the first member of the members of PARP's family proteins identified. PARP 1 is a multi-domain protein: the amino-terminal DNA-binding domain (DBD) contains zinc finger domains and a bipartite nuclear localization signal (NLS); the auto-modification domain (AD), and the carboxyl-terminal PARP homology domain, which includes the catalytic domain (CAT) responsible for PAR formation. The damage in DNA is recognized by PARP1 through zinc fingers FI and FII domains. PARP1 early recognizes nicked DNA and organizes the recruitment of the different repair systems, accumulating PAR protein targets. PARP1 is itself the major acceptor of PAR, used to attract and assist the assembling multi-protein complexes in chromatin remodeling, DNA repair and damage checkpoint signaling [22, 23]. DNA repair scaffolds as XRCC1 may be directly recruited by auto-modified PARP1. PARP1 also recruits the DNA check-point protein ATM activating the signaling cascade for DNA damage and cell cycle arrest.

NER system acts by several enzymes, including XPA, RPA, XPG, TFIIH. Among them there are endonucleases, DNA helicases, DNA synthesis and ligation enzymes. NER system can be divided into global repair and transcription-coupled repair (TCR). The last one specifically aims to repair lesions of transcribed genes.

Similarly there are two main pathways regulating DSB repair: homologous recombination repair (HRR), and non-homologous end-joining (NHEJ). NHEJ may be activated during G1-phase acting without an homologous template to guide repair process. Therefore it's defined as an “error-prone” system which often causes the occurrence of further mutations.

NHEJ includes a multistep reaction starting with both Ku 70 and Ku 80 binding proteins which rapidly constitutes a complex with DNA-dependent kinase and ligase proteins, in order to repair the breaks at DNA ends.

Recent studies have shown a determinant role of NHJE systems in the repair to DNA damage caused by radiomimetic drugs, ionizing radiation (IR), or ultraviolet (UV) light, suggesting that the recruitment of an “error-prone” system, like NHJE, may increase the cells' genetic instability [24].

HRR is the most accurate mechanism among the DSBs during S - G2 phases because the source of information is represented by the sister chromatids which are used like a template to repair the damaged DNA. HRR is introduced mainly by the so-called MNR complex, including MRE11, RAD50, and NBS1 proteins, which regulates the generation of single strand ends of DNA (ssDNA), at DSB site, thus mediating the ATR recruitment. Furthermore MNR-complex interacts with ATM, playing a crucial role for the beginning of HRR cascade, by the activation of CHEK1, CHEK2, P53, NSB1, and BRCA proteins and downstream signaling pathways [25]. The HRR mechanism begins with 5′ to 3′ end-processing at broken ends of the DNA fragments which need to be repaired. The 3′ end of ssDNA, arised by exonuclease, are stabilized, through the intervention of RAD (RAD 51, RAD52) and RAP proteins. BRCA2 then mediates the substitution of RAP1 with RAD51, and leads the pre-synaptic filament towards the homologous DNA template. Following the complementary strands pairing the DNA polymerase can extend the DNA strand, finally leading to the repaired heteroduplexed DNA which is characterized by the formation of the “Holliday junctions”.

HRR defective seems to be a major contributor to tumorigenesis in individuals carrying ATM, CHEK2, RAD51, MNR, and germline and somatic mutations of BRCA, defining a tumor cells phenotype known as homologous recombination deficiency (HRD).

The crucial role of the breast cancer susceptibility proteins, BRCA1 and BRCA2 in the DNA repair has been known since 1995 [26]. These proteins are considered the product of two distinct tumor suppressor genes which play a crucial role in the response to cellular stressors and in the activation of DNA repair processes. BRCA1 and BRCA2 germline mutations predispose to develop breast and ovarian cancer and also increases the risk to develop other cancer types including pancreatic cancer, prostate cancer, and melanoma [27–29].

In details, BRCA1 protein provides to: G1-S and G2-M transition checkpoints, transcriptional regulation and chromatin remodeling. BRCA1 is rapidly phosphorylated after DNA damage in dividing cells by different kinases. The different roles of BRCA1 is guaranteed by its several domains that allow to regulate various processes by specific interactions with other cellular factors. Among them are included: nuclear localization signals NLS, binding sites for proteins p53, cMyc, RB and a transcriptional repressor ZBRK1. These proteins are known as “guardian of genome” for their crucial role in cancer development prevention and cell cycle progression restoration. Another site is the binding domain to recruit the chromatin remodeling SW1-SNF system, which is able to create a free access to other factors involved in the repair mechanisms. BRCA1 fulfills its transcriptional regulation role interacting by BRCA C - Terminal binding site (BRCT), with RNA polymerase II, the complex p300-CBP transcriptional co-activating proteins, transcription regulator protein BACH1, the histone complexes - deacetylase HDAC1 and HDAC2. These activated complexes are needed to maintain chromatins relaxed and to promote gene expression through the recruitment of the basal transcriptional machinery. BRCT also favors the binding between BRCA and CtIP, an enzyme which together with MRN complex allows the beginning of HRR pathway. Recent studies suggested that a large group of cellular proteins interact with BRCA1 favoring the activity of HRR as the predominant mechanism to ensure the genomic stability. In contrast, HRR is not efficient in BRCA1 deficient cells, and the chromatin breaks are often rejoined by components of NHEJ in aberrant fusion between heterologous chromosomes [30].

As regards BRCA2 protein it seems to act directly in HRR, by interacting with RAD 51 protein into the nucleus. The interactions between BRCA2 and RAD 51 seem to be negatively regulated by cycline A-CDK2, and a recent study has shown that cycline D1-CDK4 destablished the cycline A-CDK2 complex, promoting BRCA2-RAD51 activity, and ultimately improving the HHR.

BRCA2 seems to interact also with the Fanconi's anemia protein ubiquitylation pathway, contributing to regulate protein-protein interactions and/or accessibility of repair factors to the damage sites. When FA genes are deficient the BRCA-mediated HHR doesn't work correctly and NHEJ will be recruited determining an increase of the genomic instability [31, 32].

**Table 1:** Phase II and III clinical trials investigated PARP inhibitors in breast and ovarian cancer

			Drug	RR(%)		mPFS(mo)		mOS(mo)	
Breast Cancer				BRCA+	BRCA-	BRCA+	BRCA-	BRCA+	BRCA-
	Phase II								
		Tutt A 2010	Olaparib	41.0		6,4			
		Gelmon KA 2011	Olaparib	0.0	0,0	5,5	1,8		
		Kaufman B 2015	Olaparib	12,9		3,7		11,0	
**Ovarian Cancer**									
	Phase II								
		Audeh MW 2010	Olaparib	33, 0					
		Gelmon KA 2011	Olaparib	31, 0	26,0	7,3	6,4		
		Ledermaan J 2016	Olaparib			11,2	5,6	34.9	24,5
		Coleman RL 2016	Rucaparib	80, 0	39,0	12.8	7,2		
		Coleman RL 2015	Veliparib	26, 5		8,1		19,5	
		Kumme S 2015	Veliparib	11, 8		2,3			
		Kaufman B 2015	Olaparib	31, 0		7.0		16.6	
		Domchelt SM 2016	Olaparib	34, 0					
	Phase III								
		Mirza MR 1016	Niraparib			21,0	9,3		

RR: response rate; mPFS: median progression free survival; mOS: median overall survival

A deeper understanding of the molecular pathways underlying the DNA repair mechanisms have led to the advent of a new family of targeted drugs able to inhibit PARPs proteins. The clinical development of these drugs was based on several evidences provided by preclinical studies, which attested the possibility to induce cancer cells die by “synthetic lethality”. This model consists of determining the simultaneous block of two main DNA repair pathways in the same cancer cell, ultimately inducing cell death by apoptosis. The first study in xenograft models has shown that once inactivated by inhibitors, PARP1 lost the ability to interact with the single strand repair system preventing DNA repair *via* the BER pathway. The accumulation of SSB leads to the collaps of the replication forks translating into DSB, which if not immediately repaired by HRR system, results into the cell death. This is precisely what happened in cancer cells defective for HRR pathways, as a result of BRCA1/2 mutations [33–35]. Indeed, in absence of a functional HRR system, these lesions are usually repaired by alternative error-prone pathways, such as NHEJ and single strand annealing (SSA) resulting in gross genomic instability and ultimately leading to the cell death.

However all the potential interactions between PARP1 and HRR pathway, have not been well-elucidated [36]. Experimental data suggest that PARP inhibition increases spontaneous HRR, but it had no effects on DSB-induced HRR [37]. Other studies have shown a possible relationship between PARP enzymes and BRCA proteins, likely involving BRCA2. Indeed BRCA2 contains three tandem oligonucleotide oligosaccharide binding folds (OB-folds ) involved in DNA binding during DNA DBS repair. This domain recognizes PAR and mediates the fast recruitment of BRCA2 to the DNA lesion needed for the first steps of HRR [38].

Subsequently several both pre-clinical and clinical studies confirmed that the sensitivity to PARPi is not limited to cells harboring BRCA 1/2 mutations, but occurs also in cells carrying mutated genes encoding other proteins involved in HRR system. The hypermethylation of the BRCA1 promoter or the loss of function of other genes as *ATM*, *CHEK2*, *RAD51*, *BRIP1*, and *PALB2* belonging at DNA repair machinery, define a specific phenotype with features and behavior similar to *BRCA*-related cancers, defined as “BRCAness”. These patients might benefit from platinum-based therapies and/or PARP inhibition like BRCA mutated [39–42].

## CLINICAL DEVELOPMENT OF PARP INHIBITORS

To date there are about ten molecules, including olaparib, veliparib, rucaparib, niraparib, among those in more advanced stages of experimental development. The majority of such studies have been focused on solid tumors harboring germ-line BRCA1-2 mutations, mainly ovarian and breast, but also prostate or pancreatic cancers. However the use of PARP inhibitors as single agent was extended also to “BRCA-like” sporadic tumors with suspected homologous recombination (HR) genes defects, including high grade serous ovarian cancer (HGSOC) and triple negative breast cancer (TNBC). Furthermore the peculiar mechanisms of action of such compounds has led to evaluate potential combinations with both DNA damaging cytotoxic agents, including chemotherapy and radiotherapy, and targeted agents able to induce HR dysfunctions, such as CDK1, PI3K, PTEN and HSP90 inhibitors or anti-angiogenic drugs.

### Ovarian cancer

Fong et al have has first demonstrated the activity of single agent PARP inhibitor olaparib in BRCA-deficient advanced ovarian, breast and prostate cancers [43], with grater activity observed in platinum sensitive ovarian cancer patients [44]. Response rates of 30% have been reported in a subsequent phase II studies conducted in refractory advanced BRCA-mutant ovarian cancer [45], suggesting BRCA1-2 mutations as potential predictive biomarkers for clinical use. However another phase II study including both BRCA mutant and wild type patients with breast cancer and HGSOC showed encouraging activity also in the cohort of wild type, platinum-sensitive HGSOC patients [46], likely due to the acquired defects of HR genes responsible for “BRCA-like phenotype”, which conferred the same sensitivity to both platinum-chemotherapy and PARP inhibition. All such findings have led to the design of a randomized phase III, placebo controlled trial, of olaparib as single agent maintenance therapy in patients with recurrent HGSOC who responded to prior platinum-based chemotherapy, showing a significant improvement of 3.6 months progression free survival (PFS) in the overall population (HR: 0.35, *p* < 0·001), with the greatest increment of 6.9 months PFS (HR 0.18, *p* < 0.0001) occurring in the subgroup of patients with BRCA mutations [47]. The updated analysis of the study 19 has recently shown a significant overall survival benefit limited to the BRCA mutant patients (34.9 *vs* 30.2 months; HR: 0.62, *p* = 0.025), which was not extended to the wild type population (HR 0.83, *p* = 0.37). As expected adverse events like fatigue, anemia, nausea and vomiting were significantly higher with olaparib than placebo [48]. On the basis of such positive results olaparib was the first PARP-inhibitor receiving the approval by the European Medical Agency (EMA) at doses of 400 mg twice daily as maintenance therapy for platinum-sensitive patients with advanced HGSOC, fallopian tube, or primary peritoneal cancer, harboring BRCA-mutations. A companion diagnostic test has been also approved by FDA to identify mutations in BRCA1/2 genes using DNA obtained from a blood sample. Along with olaparib, several other PARP inhibitors, including veliparib, rucaparib and niraparib have shown encouraging activity and acceptable safety profile in early phase I-II studies. In particular the phase II randomized ARIAL 2 study of rucaparib has shown an objective response rate (ORR) of 80% and median PFS of 12.8 months in BRCA-mutant platinum sensitive patients with recurrent ovarian cancer and ORR 39% with and median PFS of 7.2 months in BRCA wild type patients with a BRCA-like signature, compared to ORR of 13% and median PFS of 5 months in biomarker negative patients. Rucaparib was associated with a manageable safety profile, including nausea, asthenia/fatigue and ALT/AST elevations among the most common treatment-related AEs [49, 50]. These impressive results led to the recent Breakthrough Therapy designation status of rucaparib by the FDA for the treatment of ovarian cancer, while the ARIEL3 randomized study is currently recruiting patients. Veliparib has recently shown a significant activity and tolerable safety profile as single agent in a phase II single arm trial including ovarian cancer patients carrying a germline BRCA1-2 mutation who progressed to prior chemotherapy regimens, reporting ORR of 35% and 20% in platinum-sensitive and platinum-resistant patients, respectively [51]. A phase 3 trial is currently ongoing in order to further elucidate the potential of this drug in such setting. Niraparib 300 mg/day has shown a good safety profile and a promising activity with ORR 40% in pre-treated ovarian cancer patients with BRCA 1-2 mutations [52]. The phase III randomized ENGOT-OV16/NOVA trial investigated the PARP inhibitor niraparib as single agent maintenance therapy in patients with recurrent, platinum sensitive HGSOC, stratified by BRCA-mutation status. The study has met its primary end-point showing a significant PFS improvement both in BRCA-mutant (HR: 0.27; *p* < 0.001) and in BRCA-wild type (HR: 0.45; *p* < 0.0001) populations. A further analysis of BRCA-wild type patients identified the subgroup of HRD-positive patients who receive more benefit (HR: 0.38; *p* < 0.001) compared to HRD-negative patients (HR: 0.58; *p* < 0.0226) [53]. The large benefit observed in the overall population included in the NOVA study could led to a fast approval of niraparib in the treatment of platinum sensitive recurrent ovarian cancer, regardless of BRCA-status. An alternative approach has been investigated by another phase II randomized trial comparing olaparib plus chemotherapy (paclitaxel and carboplatin), followed by olaparib single agent maintenance *versus* chemotherapy alone in platinum-sensitive recurrent HGSOC patients. The results of such trial showed that combining olaparib 200 mg twice daily for 10 days of each cycle with lower dose of carboplatin (AUC 4) plus paclitaxel, followed by olaparib 400 mg twice daily as maintenance treatment is an effective and tolerable option leading to a significant 2.6-month PFS advantage (HR 0.51, *p* = 0.0012), with the greatest clinical benefit in BRCA-mutated patients (HR: 0.21, *p* = 0.0015) [54]. The addition of veliparib to cyclophosphamide didn't improve RR and PFS in patients with recurrent HGSOC [55], while another phase II randomized study comparing veliparib plus temozolomide *vs* PLD in the same setting of patients has just completed recruitment (NCT01113957, https://clinicaltrials.gov/ ). A very promising strategy emerged from the phase II randomized trial by Liu et al. which showed a further PFS benefit from adding the anti-angiogenic agent cediranib to olaparib for platinum-sensitive, recurrent, HGSOC, fallopian tube, or primary peritoneal cancer (median PFS 17.7 *vs* 9 months; (HR: 0.42; *p* = 0·005) [56], warranting investigation in a phase III ongoing trial. Similarly a phase I-II study is currently comparing tolerability and efficacy of niraparib alone *versus* niraparib-bevacizumab combination *versus* sequential bevacizumab and niraparib in platinum sensitive relapsed ovarian cancer [57], while the PAOLA1 trial is currently investigating first-line chemotherapy with bevacizumab plus olaparib or placebo as maintenance treatment. Finally the combination of olaparib and PD-L1 checkpoint inhibitor Durvalumab has shown promising durable long-term responses and a tolerable safety profile in pre-treated ovarian cancer and triple negative breast cancer (TNBC) patients [58].

### Breast cancer

As for ovarian cancer, first proof-of-concept trials have shown a significant activity of PARP inhibitors in women with advanced breast cancer and BRCA1-2 mutations [43], reporting ORR to olaparib nearly to 40% and median PFS 5.7 months in the overall population and ORR of 54% in TNBC patients [59]. Simultaneously another phase II study including both BRCA mutant and wild type patients with breast cancer and HGSOC showed no confirmed clinical responses in breast cancer patients according to RECIST criteria but only a partial reduction in tumor size for 5 (50%) of patients with BRCA mutations [46]. Recently the results of a multicenter phase II study confirmed that encouraging responses to olaparib were observed across different tumor types associated with germline BRCA1/2 mutations including breast, ovarian, prostate, and pancreatic cancer who received prior chemotherapies, and AEs most frequently reported were fatigue, nausea, and vomiting [60]. All these evidences have led to the design of phase III Olympia trials which are currently investigating the activity of olaparib as single agent both in metastatic and adjuvant treatment of breast cancer patients with germ-line BRCA1-2 mutations. Rucaparib has shown preliminary activity in a phase II study including patients with BRCA1-2-mutated metastatic breast cancer [61], and it will be early under evaluation in advanced breast cancer patients with a BRCA-like profile, including a specific genomic signature or somatic BRCA mutations (NCT02505048, https://clinicaltrials.gov/). However the addition of rucaparib to cisplatin failed to improve the DFS in patients with TNBC who had residual disease after preoperative chemotherapy [61]. Recently early phase I-II studies investigated both tolerability and activity of veliparib in combination with different chemotherapy regimens, including temozolomide, carboplatin, irinotecan, cisplatin, vinorelbine and cyclophosphamide in pre-treated advanced breast cancer patients with or without BRCA mutations, showing RR between 20% and 40%. Veliparib is currently under investigation in two phase III randomized trials comparing veliparib plus carboplatin-based regimens both in advanced and neoadjuvant setting, including BRCA-mutated and TNBC, respectively (NCT02163694; NCT02032277, https://clinicaltrials.gov/). Niraparib 300 mg/day has shown a good safety profile and a promising activity with ORR of 50% in pre-treated breast cancer patients with BRCA1-2 mutations [52], and is currently under investigation as single agent in the phase III randomized BRAVO trial in this setting of patients. Finally talazoparib is a PARP inhibitor currently under investigation as single agent in BRCA mutated breast cancer both in a phase III study in metastatic disease and in a pilot phase II study as neoadjuvant treatment (NCT02282345, https://clinicaltrials.gov/).

### Other malignancies

According to the first evidences reported by Fong et al, the results of a multicenter phase II study by Kaufman et al, have recently showed encouraging responses to olaparib across different tumor types associated with germline BRCA1/2 mutations including breast, ovarian, prostate, and pancreatic cancer who received prior chemotherapy regimens [60], confirming that BRCA1/2 germline status defines a target population responsive to PARP inhibition, regardless of tumor anatomic origin and histotype. Currently phase II-III studies are investigating the potential activity of PARP inhibitors in genetically defined advanced solid tumors harboring BRCA1/2 mutations. Particularly the phase III randomized POLO study is evaluating single agent olaparib as ‘switch maintenance' therapy in patients with BRCA1/2 mutated advanced pancreatic cancer who have not progressed on first-line platinum chemotherapy (NCT02184195, https://clinicaltrials.gov/). However, except for ovarian and breast cancers, BRCA1/2 germline mutations are less frequently observed in other malignancies, including gastric and pancreatic cancers (5%-10%), melanoma (5%) [62], prostate and NSCLC (1%-2%) [63, 64]. Beyond BRCA1/2, anti-tumor activity of PARP inhibitors was also reported in sporadic (BRCA1/2 wild type) cancers with BRCA-like phenotype. Recently a phase II study by Mateo et al. has shown promising antitumor activity of olaparib in patients with sporadic advanced CRPC who progressed to prior anticancer treatments and who had DNA-repair genes defects identified by NGS, accounting for about 25-30% of all sporadic CRPC patients [65]. Another phase II study is currently evaluating olaparib plus abiraterone in advanced sporadic CRPC including the assessment of activity in ETS fusion-positive tumors, which represent about 50% of all CRPC patients, in order to demonstrate its predictive role for clinical setting (NCT01972217, https://clinicaltrials.gov/). Similarly clinical studies are investigating the activity of PARP inhibitors in Ewing's sarcoma with either EWS-FLI1 or EWS-ERG genomic fusions. Since pre-clinical studies demonstrated the potential role of PARP inhibitors as chemo/radio-sensitizers several conducted/ongoing studies investigated the addition of such agents to chemotherapy regimens in different sporadic tumor types. Interesting activity has been recently observed with veliparib plus chemotherapy combinations both in melanoma patients with unknown BRCA/HR status [66, 67], and in untreated NSCLC patients with squamous histology, reaching an hazard ratio of 0.50 for PFS, and 0.71 for OS [68], and leading to a randomized phase III ongoing study in this subgroup of patients (NCT02264990, https://clinicaltrials.gov/), while the addition of iniparib to first-line platinum-gemcitabine did not improve ORR in advanced NSCLC patients [69]. The addition of olaparib to paclitaxel as second-line therapy in patients with recurrent/metastatic gastric cancer was well tolerated and showed a statistically significant improvement in OS in the overall included population, with a larger benefit in the subgroup of patients with low ATM expression [70], and is currently under investigation in a phase III randomized study (NCT01924533, https://clinicaltrials.gov/). Randomized phase II and III studies are ongoing to evaluate the efficacy of veliparib plus chemotherapy regimens as first-line treatment in advanced colorectal cancer (NCT02305758, https://clinicaltrials.gov/) and glioblastoma (NCT02106546, https://clinicaltrials.gov/), respectively, while a phase II randomized study is comparing olaparib plus gefitinib *vs* gefitinib alone in advanced NSCLC patients with EGFR activating mutations. Finally several studies are also exploring the role of PARP inhibitors in combination with radiotherapy in different tumor types, including NSCLC, head and neck cancer, esophageal cancer, and pancreatic cancer. Recently a meta-analysis including randomized controlled trials (RCTs) of PARP inhibitors in cancer has shown that such agents significantly improve the PFS of overall included population, and that such benefit was even greater in the subgroup of patients harboring BRCA 1/2 mutations, but failed to improve OS of cancer patients [12], and this need to be taken into account to optimize the design of further clinical studies.

## KNOWN MECHANISMS OF PRIMARY RESISTANCE

It has been already demonstrated that the activity of PARP inhibitors depends from the different levels of HR deficiency [71]. Studies *in vitro* and *in vivo* allowed to identify different genes involved in the HR pathway, whose alterations are responsible for different levels of HR deficiency finally influencing the sensitivity to PARP inhibition [72–75].

Certainly a well known mechanism of primary resistance to PARP inhibitors is represented by the mutation status of BRCA-1 and BRCA-2 genes. Indeed it has been shown that breast cancer cells with BRCA1/2 dysfunctions have a greater sensitivity to the PARP inhibitors because of the simultaneous ineffectiveness of both BER and HR mechanisms, respectively linked to the single (PARP inhibitors) and double (loss of function of BRCA1 and/or BRCA2) strand DNA damage, finally producing a chromosomal instability, cell cycle arrest and apoptosis [35, 71].

Another important gene involved in the HR pathway is PTEN. PTEN encodes a phosphatase protein regulating also the PI3K signaling pathway. Mutations and loss of PTEN function have been widely correlated with human carcinogenesis [76]. In glioblastoma cells, the deficiency of PTEN is of crucial importance since it contributes to an increased sensitivity to a temozolamide derivate (N-methylN'-nitro-N-nitrosoguanidine - MNNG). Indeed double strand DNA damages can't be repaired because of the deficiency PTEN function, therefore resulting in cells death by apoptosis [77, 78]. The same mechanism is responsible for an increased activity of PARP inhibitors also in endometrial or prostate cancer cells with loss of PTEN function compared to wild-type cells, suggesting a potential role of PTEN as a marker of primary resistance to PARP inhibitors [79, 80].

PI3K is another important protein involved in the resistance mechanisms, also because of its close relationship with the above mentioned PTEN signaling pathway. PI3K is a member of a family of lipid kinases, and its activation is required for the functionality of HR pathway [81]. Recent studies have shown that an increased BRCA gene down-regulation, caused by PI3K protein target inhibition, makes triple-negative breast cancer cells more sensitive to PARP inhibitors, suggesting as PI3K functioning could represent a possible mechanism involved in the occurrence of primary resistance [82, 83]. The discovery of the correlation between DNA damage and PI3K activity has also led to the investigation of different PARP inhibitors in solid tumors characterized by PI3K gene alterations, such as NSCLC and colorectal, whereas its prognostic and/or predictive role is currently under investigation [84, 85].

The ATM gene also seems to have a role in the HR mechanisms, therefore on the sensitivity/resistance to PARP inhibitors. The protein kinase ATM shares the chemical group -COOH with PI3K protein and it is particularly involved in the repair of double strand DNA damage caused by ionizing radiation. The dysfunction of such protein leads to an accumulation of DNA damage by oxidative stress. Previous studies have shown that the functionality of ATM and PARP are absolutely necessary for embryonic development of mice, while a functional deficiency of both proteins led to their death within the first few days of life[86]. Subsequently, other studies clarified that PARP1 is more active in cancer cells that, due to the lack of function of ATM, accumulated DNA damage (most represented and studied in prostate cancer, breast cancer, mantle cell lymphoma) [65, 87, 88]. Being PARP-1 and ATM both involved in DNA repair mechanisms (BER and HR respectively), the synergic action between these two proteins could explain the PARP inhibitors resistance of cancer cells leading wild type ATM [86, 89].

**Figure 1:**
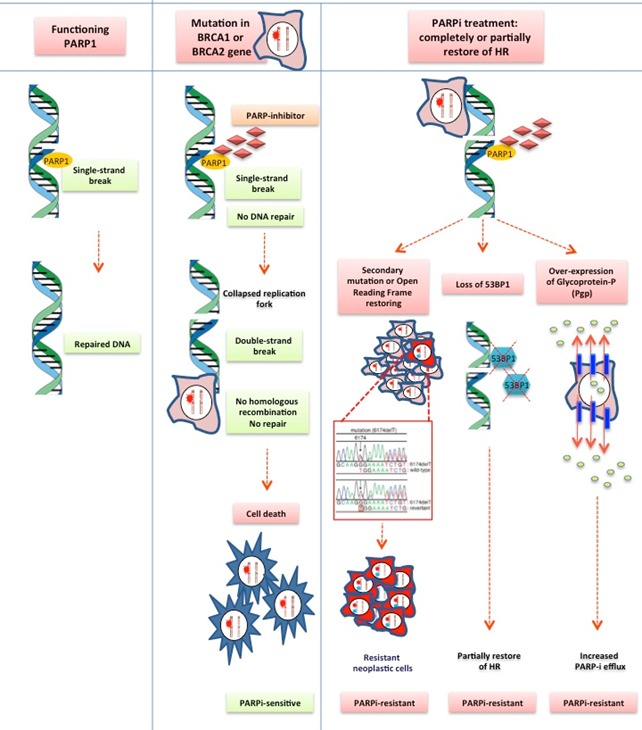
PARP inhibitor acquired resistance mechanism Based from pre-clinical studies, several alteration are responsible of partial or complete restoration of the HR repair function: secondary mutations in BRCA1-2; loss of 53BP1 protein function; Pgp over-expression and PARPi efflux.

As we know NHEJ is defective in about 40% of ovarian cancers, and a recent preclinical study has shown that it is associated with resistance to PARP inhibitors in ex vivo primary cultures independently from HR function [90] (Figure 1).

Although the validation of the above mentioned primary resistance processes is still under investigation, because of the recent advent of PARP inhibitors, it is clear how an important goal in the near future should be represented by the identification of predictive markers of response and /or resistance to PARP inhibitors to better select ideal patients to treat with these drugs. In this sense, a potential role could be played by miRNAs, fractions of non-coding RNA that regulate the post-trascriptional gene expression [91].

Indeed there are several data that suggest that miRNAs take part in modulating the cancer cells' response to PARP inhibitors. Among the most common miRNAs identified in both ovarian cancer and triple-negative breast cancer, mir-9, mir-506, mir-96, mir-182 and mir-206 are worthy of mention. Liquid biopsy could represent an ideal tool to study this interesting but at the same time greatly complex and confounding scenario [92–96].

## NEW FINDINGS ON ACQUIRED RESISTANCE

Since the advent of PARP inhibitors for cancer treatment, there are growing evidences showing that not all patients with BRCAness genes alterations report the same treatment responses. Commonly to other targeted treatments the majority of tumors will develop acquired resistance within 1 year of therapy, leading to the disease progression and the subsequent discontinuation of cancer treatment [73]. It's likely that the different emerging mechanisms of resistance may depend from the original BRCAness gene alterated, ultimately leading to different patterns of treatment response observed in clinical setting [97].

Considering the recent introduction of PARP inhibitors for clinical use, there is currently very limited understanding about the molecular mechanisms underlying the occurrence of acquired resistance to this family of drugs. The majority of available data have emerged from pre-clinical studies, showing several alterations responsible for the partial or complete restoration of the HR repair function, including: secondary mutations in BRCA1/2, Pgp overexpression, and the loss of 53BP1 protein function. Several studies have shown the occurrence of secondary mutations in the alterated BRCA1-2 genes during PARP inhibitors therapy, leading to a partial restoration of both BRCA protein and the related HR repair function, with the subsequent induction of resistance [97–99]. Mutations of C-terminal domain of BRCA1 (BRCT) may promote PARP inhibitor resistance preventing the recruitment of protein complexes which are useful in the site of the damaged DNA [100]. The same mechanism has been shown in human tumor cell lines harboring BRCA2 6174delT frameshift mutation. The result of such alteration is a truncated, non functional protein, because of the loss of C-terminal portion, BRC repeats, DNA-binding/DSS1 interaction domain, finally resulting in both platinum and PARP-inhibitors resistant phenotype [97]. The over-expression of Glicoprotein-P (Pgp) as mechanism of resistance has been first observed in ovarian cell-lines receiving combination chemotherapy treatment with Paclitaxel plus Doxorubicin. Recently a significant up-regulation of Pgp associated mdr1 a/b genes has been identified in ovarian cell lines undergone prolonged treatment with olaparib. In-vivo studies in mouse models have clearly shown as such drug may be immediately ejected by tumor cells by overexpression of Pgp on the cell surface, confirming a potential role of such protein in the development of acquired resistance to Olaparib [101]. However such kind of resistance could be overcome by the addition of Pgp inhibitors to olaparib, as well as using other members of PARP inhibitors family which are less sensitive to Pgp mechanism of action, such as veliparib.

In 2010, *in vitro* and *in vivo*-studies by Bouwman and Bounting, respectively, have first demonstrated that loss of P53-binding protein 1 (53BP1) function was associated with the partial restoration of the HR repair function in BRCA1 mutated models [102, 103]. Indeed 53BP1 protein is involved in the control of DNA clivage at DSBs mediating BRCA-mutated cells death during PARP-inhibitors treatment. Recent evidences have shown also decreased levels of 53BP1 in patients with ovarian cancer who developed acquired resistance to both platinum-based chemotherapy and PARP-inhibitors, confirming a potential role of such protein in the occurrence of resistance. Other data emerging from in vivo-studies have shown the simultaneous occurrence of different alterations responsible for the development of resistance to PARP inhibition in mouse models, including BRCA1 secondary mutations, Pgp over-expression and loss of 53BP1 protein function [102].

## CONCLUSIONS

BRCA mutations increased tumor-cells sensitivity to PARP inhibitors by synthetic lethality and several compounds targeting the PARP enzymes system have been evaluated in clinical trials. In this review we have summarized the most relevant clinical trials carried out in patients affected by advanced breast or ovarian cancer or other malignancies and treated with PARP inhibitors alone or in combination with chemotherapy. The positive results of such studies have recently led to the approval of the first PARPi olaparib as maintenance therapy for platinum-sensitive patients with advanced, recurrent HGSOC, fallopian tube, or primary peritoneal cancer, harboring BRCA-mutations. Therefore offering to test for germline BRCA1/2 mutations should now be considered a routine part of clinical practice, in order to provide the best treatment strategy to each patient. Even if BRCA1/2 mutations have shown to be highly predictive of PARPi activity in the majority of studies, PARPi have shown also activity in patients without BRCA-mutations, especially in HRD-positive population. Testing for somatic BRCA mutations and HRD-signature is currently a developing area with interesting implications for clinical practice. There are some mechanisms that have been reported as responsible of primary resistance to these molecules. BRCA wild type status, PTEN, PI3K and ATM have been evaluated as markers of primary resistance. Many studies aim to identify positive predictive markers to select patients who may benefit most from PARP inhibition. In particular it has been hypothesized that the different action of PARP inhibitors may depend on the expression of some miRNAs. All patients initially responding to PARP inhibition at some time later develop acquired resistance. The suggested mechanisms of acquired resistance include secondary mutations in BRCA1-2, Pgp over-expression and loss of 53BP1 protein function. Further pre-clinical and clinical studies are required to better define the role of these innovative targeted agents in cancer treatment. It seems reasonable that for the peculiar mechanisms of action, PARP inhibitors could be usefully combined with both DNA damaging cytotoxic agents, radiotherapy, and with other targeted therapies. Initial results of combining PARPi with anti-angiogenic drugs are promising, leading to several randomized studies in different lines of treatment. Finally PARPi and PD1/PDL1 checkpoint inhibitors combinations have recently shown durable responses, emerging as another promising strategy to expand the treatment arsenal against cancer.
